# Structural and functional determination of homologs of the *Mycobacterium tuberculosis N*-acetylglucosamine-6-phosphate deacetylase (NagA)

**DOI:** 10.1074/jbc.RA118.002597

**Published:** 2018-05-04

**Authors:** Mohd Syed Ahangar, Christopher M. Furze, Collette S. Guy, Charlotte Cooper, Kathryn S. Maskew, Ben Graham, Alexander D. Cameron, Elizabeth Fullam

**Affiliations:** From the ‡ School of Life Sciences and; the § Department of Chemistry, University of Warwick, Warwick, Coventry CV4 7AL, United Kingdom

**Keywords:** carbohydrate, crystal structure, tuberculosis, Mycobacterium tuberculosis, enzyme kinetics, X-ray crystallography, peptidoglycan, bacterial cell wall, N-acetylglucosamine-6-phosphate deactylase, TB

## Abstract

The *Mycobacterium tuberculosis* (*Mtb*) pathogen encodes a GlcNAc-6-phosphate deacetylase enzyme, NagA (Rv3332), that belongs to the amidohydrolase superfamily. NagA enzymes catalyze the deacetylation of GlcNAc-6-phosphate (GlcNAc6P) to glucosamine-6-phosphate (GlcN6P). NagA is a potential antitubercular drug target because it represents the key enzymatic step in the generation of essential amino-sugar precursors required for *Mtb* cell wall biosynthesis and also influences recycling of cell wall peptidoglycan fragments. Here, we report the structural and functional characterization of NagA from *Mycobacterium smegmatis* (MSNagA) and *Mycobacterium marinum* (MMNagA), close relatives of *Mtb*. Using a combination of X-ray crystallography, site-directed mutagenesis, and biochemical and biophysical assays, we show that these mycobacterial NagA enzymes are selective for GlcNAc6P. Site-directed mutagenesis studies revealed crucial roles of conserved residues in the active site that underpin stereoselective recognition, binding, and catalysis of substrates. Moreover, we report the crystal structure of MSNagA in both ligand-free form and in complex with the GlcNAc6P substrate at 2.6 and 2.0 Å resolutions, respectively. The GlcNAc6P complex structure disclosed the precise mode of GlcNAc6P binding and the structural framework of the active site, including two divalent metals located in the α/β binuclear site. Furthermore, we observed a cysteine residue located on a flexible loop region that occludes the active site. This cysteine is unique to mycobacteria and may represent a unique subsite for targeting mycobacterial NagA enzymes. Our results provide critical insights into the structural and mechanistic properties of mycobacterial NagA enzymes having an essential role in amino-sugar and nucleotide metabolism in mycobacteria.

## Introduction

*Mycobacterium tuberculosis* (*Mtb*)^2^ is a major human pathogen and is the causative agent of tuberculosis (TB). In 2016, there were ∼10.4 million people with new TB infections, and 1.7 million people died of the disease (1). The emergence of drug-resistant strains of *Mtb* has resulted in untreatable forms of TB, and there is therefore an urgent requirement for novel strategies to address this global health threat. The success of *Mtb* as a pathogen is due in part to the complex mycobacterial cell wall that comprises three distinct macromolecules: peptidoglycan (PG), arabinogalactan (AG), and mycolic acids that form the mAGP complex and is further interspersed with distinctive lipids and an outermost capsule of polysaccharides and proteins (2). The cell envelope has been implicated in key roles in the survival and virulence of *Mtb* and as such is an important target for current TB drugs and compounds that are in clinical development (3).

**Figure 1.**
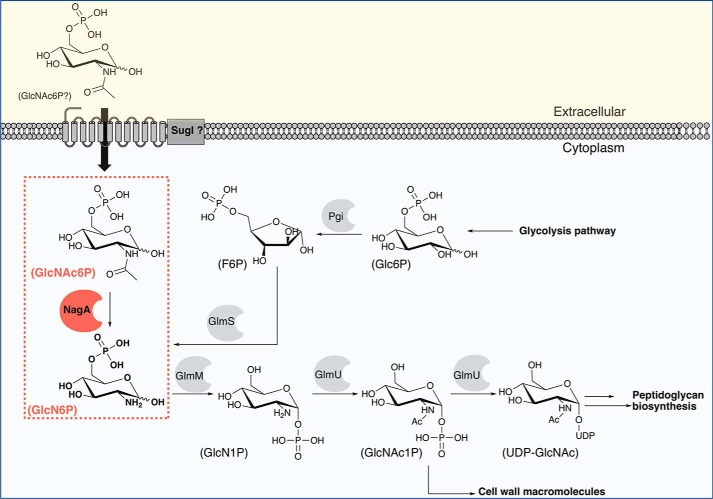
**Schematic diagram of the pathways of amino sugar metabolism in *M. tuberculosis*.** The reaction catalyzed by NagA (GlcNAc6P deacetylase) is highlighted in *red. Glc6P*, glucose-6-phosphate; *F6P*, fructose-6-phosphate; *GlcN6P*, glucosamine-6-phosphate; *GlcN1P*, glucosamine-1-phosphate; *GlcNAc1P*, GlcNAc-1-phosphate; *SugI*, integral sugar transporter; *Pgi*, glucose-6-phosphate isomerase; *GlmS*, glutamine-fructose-6-phosphate aminotransferase; *GlmM*, phosphoglucosamine mutase; *GlmU*, bifunctional acetyltransferase/uridyltransferase.

Glucosamine-6-phosphate (GlcN6P) is an essential amino-sugar that acts as a gatekeeper between the glycolysis metabolic pathway and cell wall biosynthesis (Fig. 1) (4). Glucosamine derivatives are key precursors in the synthesis of *Mtb* PG found in the *Mtb* disaccharide GlcNAc–l-rhamnose, which links PG → AG (5, 6), and are additionally important in *Mtb* resuscitation pathways (7, 8) and cell wall recycling of murein derivatives (9–13). The isomerization of GlcN6P to glucosamine-1-phosphate is the first step of *Mtb* cell wall biosynthesis (14, 15) (Fig. 1). In *Mtb* GlcN6P can be derived from two biosynthetic pathways. Either GlmS (*Mtb* Rv3436) isomerizes fructose-6-phosphate from the glycolysis pathway to GlcN6P (14, 16) or GlcN6P can be derived from deacetylation of GlcNAc-6-phosphate (GlcNAc6P) by a putative GlcNAc6P deacetylase enzyme (NagA), which is assigned to *Mtb* ORF Rv3332 (NagA) (17). Hence, the *Mtb* GlmS and NagA enzymes have key roles in controlling the intracellular pools of GlcN6P, and the *in vitro* essentiality of both genes has been established in *Mtb* from transposon mutagenesis studies (18). In addition, metabolic profiling of guinea pigs infected with *Mtb* showed up-regulation of NagA in infected lung tissue (13, 19).

NagA enzymes (EC 3.5.1.25) are widespread in nature and found in a range of eukaryotic and prokaryotic species (20) as metal-dependent enzymes with a physiological role in catalyzing the deacetylation of GlcNAc6P to yield GlcN6P and acetate (Fig. 1) (21–23). A putative catalytic mechanism for NagA enzymes has been proposed in which nucleophilic attack occurs via a water/hydroxide ion. The mechanism proceeds via a strictly conserved active-site aspartic acid residue that has an essential role in catalysis and is understood to act initially as a base to activate the hydrolytic water molecule and then as an acid to protonate the amine leaving group (23–25). High-resolution X-ray structures have been determined of NagA from three Gram-negative bacteria species: *Escherichia coli* (PDB codes 1YMY, 1YRR, 2P50, and 2P53) (21, 23) with and without a transition state inhibitor (*N*-methylhydroxyphosphinyl-d-glucosamine-6-phosphate), *Thermotoga maritima* (PDB code 1O12), and *Vibrio cholerae* (PDB 3EGJ and 3IV8); and the Gram-positive *Bacillus subtilis* organism (PDB code 2VHL) with the GlcN6P reaction product (24). All NagA structures characterized to date reveal a similar overall architecture and arrangement of two domains. Domain I comprises a (β/α)_8_-barrel structural fold that forms the dimeric interface with domain I of the neighboring subunit. This dimeric interface enables the formation of two identical active sites that are involved in substrate and metal co-factor recognition. The smaller second domain of NagA enzymes comprises a β-barrel with unknown biological function. There are intriguing differences in the active site metal-binding site. The NagA enzyme from *E. coli* contains a mononuclear metal-binding site (21–23), whereas the corresponding enzymes from *T. maritima* and from the Gram-positive *B. subtilis* both have binuclear metal-binding sites, though in the former case only one metal ion is necessary for catalysis (22, 24). It is therefore possible that NagA enzymes have evolved to have a different role or catalytic function depending on the organism in which they are found.

**Figure 2.**
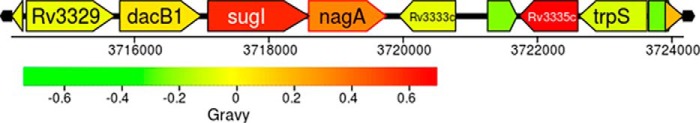
**The NagA operon in *M. tuberculosis*.** The operon organization was taken from Xbase. The accession numbers for these genes in *M. tuberculosis* H37Rv are as follows: Rv3329 (*probable aminotransferase*), Rv3330 (*dacB1*, *probable penicillin-binding protein* d-alanyl-d-alanine carboxypeptidase), Rv3331 (*sugI*, *probable sugar-transport integral membrane protein*), and Rv3332 (*nagA*, *GlcNAc-6-phosphate deacetylase*).

Within *Mtb*, NagA (Rv3332) is found in a four-gene operon also encoding a putative aminotransferase (Rv3329); dacB1 (Rv3330), an enzyme involved in PG maturation; and SugI (Rv3331), a putative sugar-importer (17, 26) (Fig. 2). Despite its importance in *Mtb*, detailed studies of NagA from mycobacteria have not yet been reported. This study focuses on the NagA homologs from *Mycobacterium marinum* and *Mycobacterium smegmatis*, close relatives to *Mtb*. Here, we describe the functional characterization and structural determination of mycobacterial NagA enzymes. We report the crystal structure of WT and mutant *M. smegmatis* NagA, both in ligand-free form and complexed to the GlcNAc6P substrate, revealing the molecular determinants and structural framework of the active site. Furthermore, we have carried out site-directed mutagenesis targeting putative catalytic residues allowing us to elaborate on the molecular recognition of NagA indicating the importance of conserved active-site amino acids in the catalytic function and substrate and stereochemical requirements of this important *Mtb* enzyme.

## Results

### Production of MMNagA and MSNagA

To produce recombinant NagA protein, the *nagA* gene was amplified by PCR and cloned into either the pET28a plasmid for co-expression in *E. coli* containing the *Mtb* GroES chaperone or pYUB1062 (27) for expression in *M. smegmatis*, both bearing an N-terminal His_6_ tag. It proved difficult to obtain the recombinant *Mtb* NagA protein, and therefore homologous enzymes from *M. marinum* and *M. smegmatis* were used in these studies, which have a high degree of sequence identity to the TBNagA enzyme with 71 and 52% sequence identity, respectively, at the amino acid level (28) (Fig. S1). Soluble, active MMNagA and MSNagA proteins were obtained and purified to apparent homogeneity using Co^2+^ affinity, anion exchange, and size-exclusion chromatography (Fig. S2 and S3). The identities of the NagA proteins were confirmed using in-gel trypsin digestion and analysis of the peptides by MS.

### Substrate specificity of MMNagA and MSNagA

**Figure 3.**
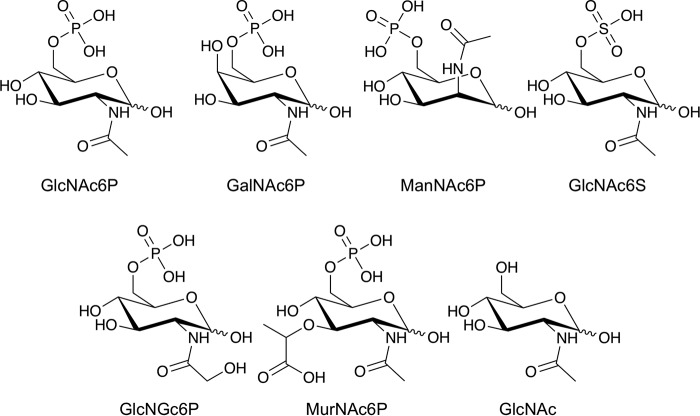
**Panel of carbohydrates probed in the kinetic studies.**

**Figure 4.**
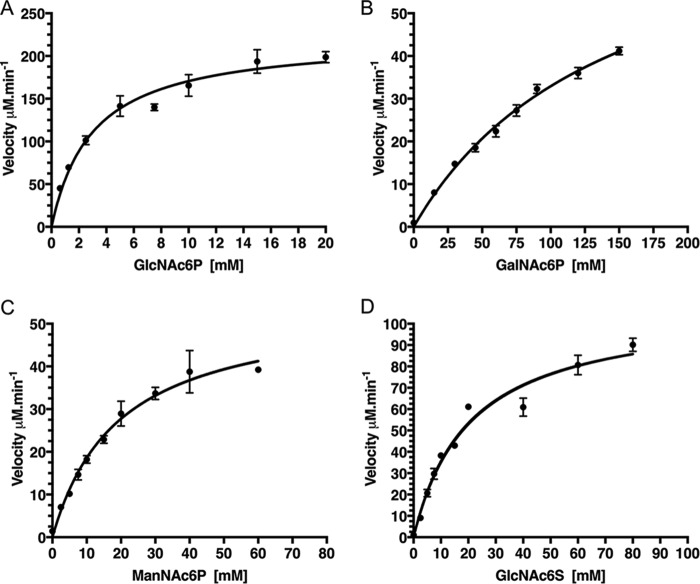
**Substrate dependence of MMNagA activity.** Michaelis–Menten curves were fitted, and selected curves are shown for MMNagA with the substrates GlcNAc6P (*A*), GalNAc6P (*B*), ManNAc6P (*C*), and GlcNAc6S (*D*). The initial velocity data were plotted against the substrate concentration, and each assay was carried out in triplicate and expressed as a value ± standard error of mean.

NagA enzymes from other species (Fig. S1), are known to catalyze the deacetylation of GlcNAc6P (20–23), and we therefore speculated that mycobacterial NagA enzymes catalyze a similar reaction. The ability of the MSNagA and MMNagA proteins to catalyze the deacetylation of various *N*-acetyl amino sugars was tested using a fluorescence-based assay by monitoring the production of the fluorescent product formed from the reaction of fluorescamine and the free-amino group that is formed following deacetylation (29). The panel of carbohydrates tested is shown in Fig. 3. We first examined the NagA reaction with various phosphorylated *N*-acetylated amino sugars that included the predicted GlcNAc6P substrate, alongside the epimeric analogs GalNAc-6-phosphate (GalNAc6P) and ManNAc-6-phosphate (ManNAc6P) (Fig. 3). The measurements indicated that MMNagA and MSNagA are active deacetylase enzymes with markedly similar kinetic profiles (Table 1). In each case we were able to detect the formation of GlcN6P, GalN6P, and ManN6P in the reaction, with both MSNagA and MMNagA showing a clear preference for the GlcNAc6P substrate (Table 1). Although MSNagA and MMNagA show activity toward GalNAc6P and ManNAc6P, kinetic analysis revealed that the *K_m_* value was 40-fold higher for GalNAc6P and 6-fold higher for ManNAc6P than for GlcNAc6P (Table 1 and Fig. 4). Intriguingly, the apparent kinetic constants varied for the MSNagA enzyme depending on whether the protein had been expressed in an *E. coli* or *M. smegmatis* host expression system with a higher *K_m_* value of 5.2 mm and almost 5-fold reduction in the *k*_cat_ found for MSNagA when expressed in *E. coli* (Table 1).

**Table 1:** **Steady-state kinetic parameters of MMNagA and MSNagA** Each assay was carried out in triplicate and is expressed as ± S.E. —, not detected/no activity.

Enzyme	Expression host	Substrate	*K_m_*	*k*_cat_	*k*_cat_/*K*_m_(mm × 10^3^)
			*mm*	*s*^−*1*^	*m*^−*1*^ *s*^−*1*^
MMNagA	*E. coli*	GlcNAc6P	3.0 ± 0.4	36.8 ± 1.6	12.3 ± 1.7
MMNagA	*E. coli*	GalNAc6P	145.4 ± 14.2	5.4 ± 0.3	0.03 ± 0.01
MMNagA	*E. coli*	ManNAc6P	19.3 ± 2.1	18.2 ± 0.8	1.1 ± 0.1
MMNagA	*E. coli*	GlcNAc6S	20.9 ± 2.4	36.0 ± 1.6	1.7 ± 0.2
MMNagA	*E. coli*	GlcNAc	—	—	—
MMNagA	*E. coli*	GlcNGc6P	—	—	—
MMNagA	*E. coli*	MurNAc6P	—	—	—
MMNagA QXN	*E. coli*	GlcNAc6P	26.7 ± 2.7	6.6 ± 0.3	0.3 ± 0.02
MMNagA AXA	*E. coli*	GlcNAc6P	—	—	—
MMNagA E127A	*E. coli*	GlcNAc6P	—	—	—
MMNagA H139A	*E. coli*	GlcNAc6P	—	—	—
MMNagA R225A	*E. coli*	GlcNAc6P	—	—	—
MMNagA H249A	*E. coli*	GlcNAc6P	—	—	—
MMNagA D272A	*E. coli*	GlcNAc6P	—	—	—
MSNagA	*E. coli*	GlcNAc6P	5.2 ± 1.0	20.7 ± 1.8	4.0 ± 0.8
MSNagA D267A	*E. coli*	GlcNAc6P	—	—	—
MSNagA	*M. smegmatis*	GlcNAc6P	3.2 ± 0.4	91.1 ± 5.5	28.0 ± 3.8
MSNagA	*M. smegmatis*	GalNAc6P	118.5 ± 10.2	31.1 ± 1.5	0.3 ± 0.03
MSNagA	*M. smegmatis*	ManNAc6P	19.1 ± 1.8	70.6 ± 2.5	3.7 ± 0.4
MSNagA	*M. smegmatis*	GlcNAc6S	21.4 ± 2.1	33.2 ± 1.3	1.6 ± 0.2
MSNagA	*M. smegmatis*	GlcNAc	—	—	—
MSNagA	*M. smegmatis*	GlcNGc6P	—	—	—
MSNagA	*M. smegmatis*	MurNAc6P	—	—	—
*E. coli* NagA	*E. coli*	GlcNAc6P	5.2 ± 1.1	97.2 ± 5.3	18.7 ± 0.05

The importance of the phosphate group in substrate recognition and catalysis was examined through the reaction of NagA with GlcNAc-6-sulfate (GlcNAc6S) and GlcNAc. MMNagA and MSNagA were both able to catalyze the reaction with GlcNAc6S; however, in both instances the *K_m_* values were 7-fold higher (Table 1 and Fig. 4) than the phosphorylated analog. No catalytic activity toward GlcNAc was observed for either enzyme, indicating the importance of an anionic group at the 6-position and, in particular, a preference for the phosphate group for substrate recognition and catalysis. Next, we determined the importance of the *N-*acetyl group position at C2 through replacement with an *N-*glycolyl group, which can be tolerated by mammalian NagA homologs (30); however, only low levels of enzymatic activity for both MMNagA and MSNagA enzymes with this derivative with an extended linker at the C2 position were observed. Finally, we determined whether the mycobacterial NagA enzymes could act as a deacetylase for *N*-acetyl muramic acid 6-phosphate, which incorporates a C3 lactoyl group into GlcNAc6P, given that *N*-acetyl muramic acid is the main other sugar constituent of the peptidoglycan backbone. However, no detectable catalytic activity was observed in this instance indicating selectivity of mycobacterial NagA enzymes for the GlcNAc6P cell wall peptidoglycan fragment.

### Metal dependence of MMNagA and MSNagA

NagA homologs from other organisms require a divalent metal ion for catalysis (20–23). To further determine the metal content of MMNagA and MSNagA enzymes, protein samples were analyzed for the metals: manganese, iron, cobalt, nickel, copper, zinc, and cadmium using inductively coupled plasma MS (ICP–MS). From these studies, it was clear that when both MMNagA and MSNagA enzymes were expressed using an *E. coli* expression system in either LB medium or terrific broth, both zinc and iron were the predominant metals and were found in a ∼1:2 zinc:iron ratio (Table S2). In comparison, when the MSNagA enzyme is expressed using a *M. smegmatis* expression system in LB medium, the protein contained predominantly more zinc in a 12:1 zinc:iron ratio. These differences in the selected metal ion that is incorporated into the protein during protein production may explain the increase in the *K_m_* and reduction in *k*_cat_ for the MSNagA protein that is expressed in an *E. coli* host system (Table 1). Expression of MSNagA in *E. coli* in LB medium supplemented with 1 mm ZnCl_2_ did not alter the zinc:iron ratio, suggesting that the host expression system plays a significant role in metal co-factor selectivity.

CD spectroscopy revealed that removal of the metal ions, confirmed by ICP–MS, from either enzyme by extensive dialysis against chelex-treated buffer containing 10 mm 1,10-phenanthroline and 10 mm EDTA, resulted in unfolding and loss of catalytic activity (Fig. S4). Activity could not be restored by addition of ZnCl_2_ to the metal-free enzymes, indicating that the metal ions are tightly coordinated in the active site and have an essential structural role in mycobacterial NagA enzymes.

### Analysis of the pH dependence of MMNagA and MSNagA

To investigate the pH optimum for NagA catalysis with GlcNAc6P, the specific activities of the MMNagA and MSNagA enzymes were determined with GlcNAc6P across a range of pH values (Fig. S5). This shows the loss of catalytic activity at a pH below 5.5. The pH profile resembles that found for the *E. coli* NagA enzyme (23), where deprotonation of a catalytic acidic aspartic acid residue is necessary for activity.

### Site-directed mutagenesis of MMNagA and MSNagA

To assess the significance of selected MMNagA and MSNagA residues in molecular recognition and function, we generated single point mutations in five amino acids in MMNagA, with one corresponding mutation in MSNagA, that were expected to form either the ligand- or metal-binding sites. In addition, we generated two point mutations in the binuclear metal binding site of the MMNagA enzyme. The H*X*H motif (His-56–His-58), also found in *B. subtilis* and *T. maritima* NagA enzymes, was mutated to either a Q*X*N motif to replicate the single metal-binding motif found in both the *E. coli* and *V. cholerae* NagA enzymes, or to A*X*A. For each mutant, we confirmed that the introduced mutation was not detrimental to the correct folding of the protein by CD spectroscopy (Fig. S4).

In each case the single amino acid mutation resulted in complete loss of activity of the MMNagA and MSNagA proteins (Table 1) under the conditions tested, supporting the notion that these residues have a key influence on substrate selectivity and/or catalysis. To ensure that the mutation did not result in reduction/loss of metal ion during expression and/or purification, zinc was added to the reaction mixture, but this did not restore activity. Increasing the concentration of GlcNAc6P substrate and protein along with the reaction time did result in the observation of low (<5%) residual activity for three MMNagA mutants: H139A, H249A, and R225A compared with WT. The Q*X*N and A*X*A double mutants in the second metal-binding site motif resulted in protein that, although correctly folded, was inherently less stable. However, catalytic activity for the MMNagA Q*X*N mutant with GlcNAc6P was observed, with a 7-fold increase in *K_m_* value compared with WT MMNagA (Table 1). In contrast, no activity was detected for the A*X*A MMNagA mutant with GlcNAc6P. Despite these mutations in the active site of MMNagA and MSNagA being detrimental for the catalytic activity of these enzymes, the mutants were still able to bind and recognize the GlcNAc6P substrate, as evidenced from microscale thermophoresis (MST) studies (Table S3).

### Overall structure of MSNagA

To determine the molecular mechanism and structural basis of catalysis, we solved the crystal structure of MSNagA with and without the GlcNAc6P substrate present. The MSNagA protein in the absence of ligand crystallized in space group P1 with four molecules in the asymmetric unit. Phases for the structure of ligand-free MSNagA were determined by molecular replacement using the structure of *E. coli* NagA (PDB code 2P50) as a search model, and the structure was refined at a resolution of 2.6 Å. To investigate the interaction of the GlcNAc6P substrate with the protein, we mutated Asp-267, which has been shown to be important for catalysis in other NagA enzymes (21–24), to alanine. This resulted in significant loss of enzymatic activity (Table 1) and enabled co-crystallization of the MSNagA enzyme in the presence of GlcNAc6P. The crystals grew under identical conditions to the native protein with crystals in space group C2. The structure was solved by molecular replacement using the *apo*-MSNagA as a model and refined at a resolution of 2.0 Å. The refined structures have *R*_work_ values of 24.7% (*apo*; PDB code 6FV3) and 18.4% (ligand-bound; PDB code 6FV4), with *R*_free_ values of 29.5 and 23.7% respectively. Statistics of the diffraction data and structure refinement are summarized in Table 2.

**Table 2:** **Crystallographic parameters for the MSNagA and GlcNAc6P–MSNagA complex**

	*apo*-MSNagA	GlcNAc6P–MSNagA
**PDB ID**	6FV3	6FV4

**Data collection**		
Beamline	Diamond I04	Diamond I24
Wavelength (Å)	0.9795	0.9686
Space group	*P1*	*C2*
Unit cell parameters		
*a* (Å)	60.45	164.76
*b* (Å)	86.39	54.43
*c* (Å)	89.93	110.26
α (°)	88.12	90.00
β (°)	75.58	123.22
γ (°)	69.79	90.00
Molecules in ASU	4	2
Resolution, (outer shell) (Å)*^a^*	55–2.58 (2.62–2.58)	80.14–1.97 (2.08–1.97)
Unique reflections	51,044 (3,798)	55,882 (8,181)
Multiplicity	2.6 (2.7)	6.5 (6.30)
*CC*_1/2_	0.960 (0.434)	0.993 (0.841)
Completeness (%)*^a^*	98.2 (98.0)	96.8 (98.1)
*R*_merge_ (%)*^a^*	17.3 (90.2)	12.8 (82.2)
Mean *I*/σ(*I*)*^a^*	4.3 (1.0)	9.1 (2.7)

**Refinement**		
*R*_work_ (%)	24.7	18.4
*R*_free_ (%)	29.5	23.7
r.m.s.d.		
Bond lengths (Å)	0.005	0.007
Bond angles (°)	1.0	0.91
No. of nonhydrogen atoms		
Protein atoms	10,714	5,513
Ligand/ions	8	35
Solvent waters	333	530
Average B factors (Å^2^)		
Overall	42.7	37.4
Protein	42.9	36.9
Ligand/ions	40.7	46.7
Solvent	34.5	42.1
Ramachandran plot*^b^*		
Favored region (%)	94.3	96.33
Allowed region (%)	4.75	3.2
Outer region (%)	0.95	0.39

*^a^* The numbers in parentheses refer to the highest-resolution shell.

*^b^* Ramachandran plot statistics were calculated by MolProbity.

**Figure 5.**
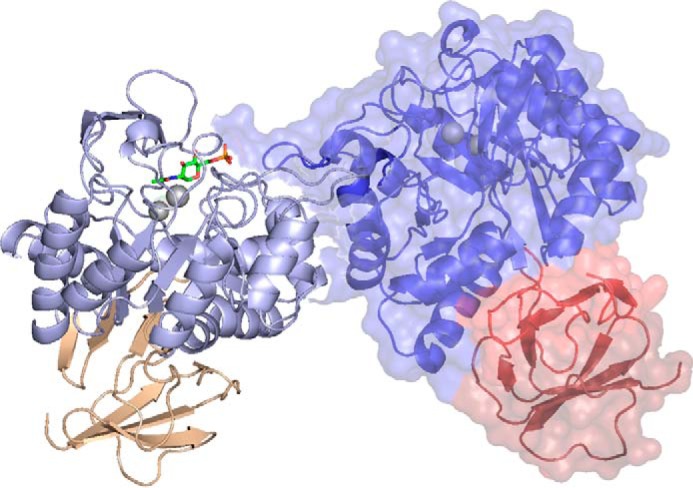
**Crystal structure of MSNagA.** Shown is MSNagA structure with one subunit (chain A) represented as a cartoon and the other subunit (chain B) with surface representation. Domain I is colored *blue*, and domain II is colored *brown* (chain A)/*red* (chain B)). The metal ions are represented as *gray spheres*, and the GlcNAc6P ligand is shown in *stick* representation.

Overall, the structure of MSNagA is very similar to other NagA enzymes with NagA from *E. coli*, the closest structural match according to PDBeFOLD (31), aligning with a root mean square deviation of 1.4 Å for 334 aligned residues (target residues, 354; sequence identity, 35%; PDB code 3IV8). The structure can be divided into two domains. Domain I (residues 52–344) is a twisted (β/α)_8_-TIM barrel that is comprised of eight alternating β-stands and α-helices and encloses the catalytic site of the enzyme and the metal-binding site. Domain II is a small β-barrel comprising eight β-strands contributed from the N and C termini of the MSNagA protein (amino acids 1–51 and 344–382) (Fig. 5). In both of the MSNagA crystal structures, the protein forms a homodimer (Fig. 5) burying a total of 3,200 Å^2^ of the surface area (32). By comparison to the determined crystal structures of NagA enzymes, this dimeric interaction is similar across bacterial NagA enzymes (21, 22, 24). The two active sites of the MSNagA homodimer are formed at the interface of domain I between the two monomeric subunits (Fig. 5) with hydrogen bonding between residues on α_8_ and α_9_ and salt bridges between Glu-230 and Arg-251. A long flexible loop formed from residues 203–223 of one subunit extends out to α_9_ of its neighboring subunit (residues 248–258), providing additional stability at the dimer interface. In solution, MMNagA and MSNagA are found as dimers as shown by size exclusion chromatography (Figs. S2 and S3), and it is likely that this dimeric oligomerization state is also the biologically relevant unit for mycobacterial homologs, which has been found for other NagA enzymes (21, 22, 24, 33).

### The ligand-binding site of MSNagA

**Figure 6.**
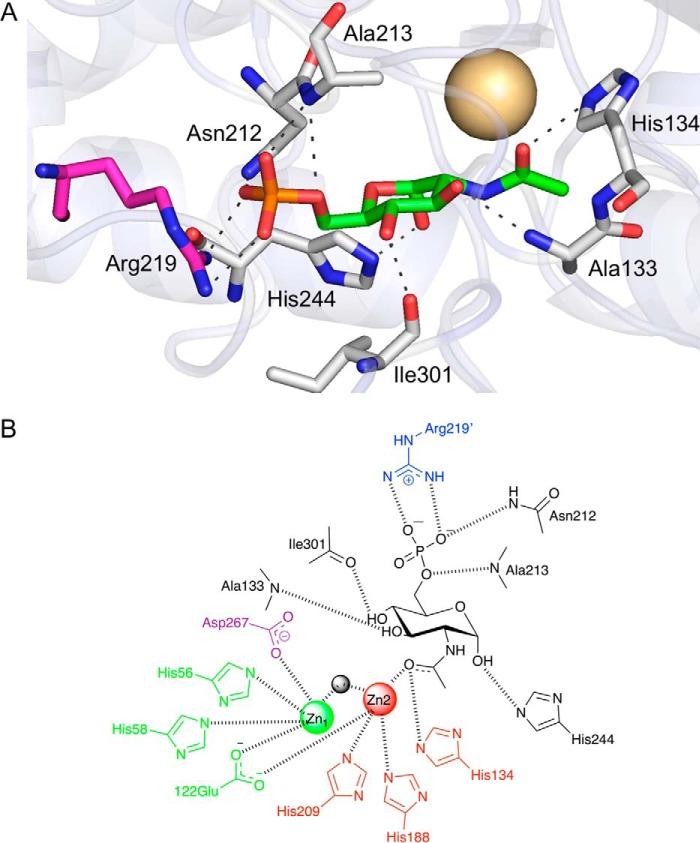
**The GlcNAc6P substrate-binding site in MSNagA.** *A*, illustration showing GlcNAc6P with *green* carbon atoms, the metal ion as a *pale-yellow sphere*, and selected amino acid residues in *stick* representation (colored *gray* for chain A, and *magenta* for chain B). *B*, schematic diagram of the interactions of MSNagA with GlcNAc6P. The residues that interact with the M1 metal are shown in *green*, the residues that interact with the M2 metal are shown in *red*, the residues that interact with the GlcNAc6P substrate are shown in *black*, the residues that interact with the GlcNAc6P substrate from the opposing MSNagA monomer are shown in *blue*, and the Asp-267 residue that is mutated in the ligand-bound structure is shown in *purple*.

From our MST studies (Table S3) the D267A MSNagA mutant was still able to interact with the GlcNAc6P substrate, although no catalytic activity was observed (Table 1). The co-crystal structure shows clear electron density for GlcNAc6P in the substrate-binding pocket of Chain A, enabling the substrate to be modeled in the active-site pocket at the dimeric interface (Fig. 6 and Fig. S6). However, it may be that the site is not fully occupied, and no electron density for the GlcNAc6P substrate was observed in the second active site of chain B where instead a flexible loop region from Glu-122 to Pro-136 partially occludes the active site (Fig. S7). The reasons for these differences between the equivalent active sites in the co-crystallized complex are not clear; however, it is intriguing that a similar situation is also observed in the co-crystal structure of the enzyme from *B. subtilis* with the GlcN6P reaction product, where only partial occupancy (<0.3) was observed in the second binding pocket (24).

The GlcNAc6P substrate is situated in the cavity of the (α/β) barrel of domain I at the dimer interface, recruiting Arg-219 from the opposing monomer through an interaction with the phosphate moiety. An arginine at this position is specific to MSNagA and in both *M. marinum* and *Mtb* NagA homologs the equivalent residue is replaced by a histidine residue (Fig. S1). The side chain of the conserved arginine residue corresponding to Arg-220 in MSNagA points away from the GlcNAc6P ligand (Fig. S8).

The GlcNAc6P substrate is found in the α-configuration and is anchored in place through the formation of hydrogen bonds with the side chains of His-244 that interacts with the anomeric GlcNAc6P hydroxyl group and with Asn-212 to the phosphate group. The remaining hydrogen bonds are through interactions with the main chain, with the equatorial C3-hydroxy group interacting with Ala-133 and the equatorial C4-hydroxy with Ile-301. These critical residues that interact with GlcNAc6P are conserved in homologous NagA enzymes with the exception of Ile-301 (MSNagA), which is instead replaced by a leucine residue; however, this modification is unlikely to affect its interaction to the substrate with the interaction occurring through its backbone carbonyl group. Additional interactions of the phosphate group are with the backbone amino group of Ala-213 and the guanidine group of Arg-219 from the opposing molecule (Fig. 6).

The carbonyl group of the GlcNAc6P substrate is positioned ∼2.5 Å from the M1 metal ion, which is ideally primed to stabilize the transition state intermediate formed during catalysis (Fig. 6). It is of interest to note, by comparison of the *apo*- and ligand-bound structures, that there is little conformational change in the binding pocket upon substrate binding to MSNagA. However, there is a difference in two loop regions that impact on the accessibility of the active site. The surface-based loop that comprises the amino acids Met-274–Ser-304 forms a dynamic “lid” that is likely to be functionally important in facilitating ligand binding through an opening/closing mechanism. An additional loop that differs between the two structures, comprising residues Glu-122–Pro-136, partially occludes the active site in chain B and prevents substrate binding (Fig. S7). Although domain II is not involved in substrate binding, we do observe a change in the position of the β-barrel in chains A and B of the ligand-bound structure.

### Binding of metal ions to MSNagA

**Figure 7.**
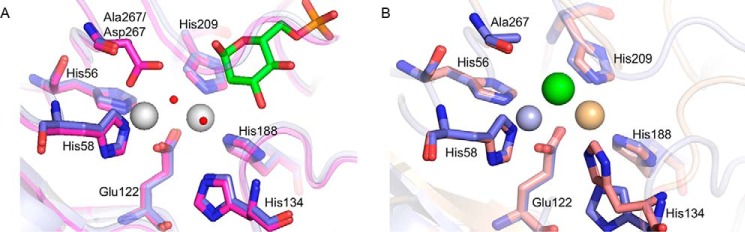
**Structure of the metal-binding site of MSNagA.** *A*, illustration showing metal-binding site in *apo*-MSNagA structure (chain A, *magenta*) superposed with ligand-bound structure (chain A, *blue*). *Silver spheres*, Zn^2+^; *red spheres*, H_2_O. GlcNAc6P with *green* carbon atoms and selected amino acid residues are shown in *stick* representation (colored by *apo* (*magenta*) or ligand-bound (*blue*)). *B*, superposition of the metal-binding site of chain A and chain B of the ligand-bound structure. *Light blue*, chain A; *light brown*, chain B; *silver spheres*, Zn^2+^; *orange sphere*, Cd^2+^; *green sphere*, Cl^−^. Selected amino acid residues are shown in *stick* representation (colored by chain A (*blue*) or ligand-bound (*salmon*)).

Although no divalent cations were added during the purification steps, well defined electron density corresponding to two bound metal ions was clearly visible in the cavity of the (α/β) barrel buried at the bottom of the active-site cleft (Fig. 7). Each MSNagA monomer in both the ligand-free and ligand-bound models contains two metal ions positioned between 3.1 and 3.4 Å apart from each other. The proteins were shown to contain both zinc and iron in the ICP–MS analysis (Table S2). Given that the same protein expressed in *M. smegmatis* contains predominantly zinc, it was decided to model the metal-binding sites as zinc rather than iron (see “Experimental procedures”; Fig. S9). However, for the ligand-bound mutant structure, residual density in the M1 metal-ion binding site indicated a larger ion. We therefore modeled a cadmium ion at this position, which is likely to arise as a result of the addition of CdCl_2_ in the crystallization conditions with the metal-binding site perhaps being destabilized by the D267A mutation.

All of the ligands that interact with the metal ions are contributed by conserved residues from one subunit of the dimer. In all chains of the *apo*-structure, the metal ion that is more buried (M1) makes four interactions coordinated by the side chains His-56, His-58, Glu-122, and Asp-267 (Fig. 7). The second metal ion (M2) is held in position by the side chains of residues His-188, Glu-122, and His-209 (Fig. 7). Coordination is completed by water molecules with one water molecule modeled as bridging both of the metals and another bound to M1. In the ligand-bound structure, the water molecule that bridges the two metal ions in the *apo*-structure is instead replaced with a chloride ion. The coordination of the metal ions in chain A to His-56, His-58, and Glu-122 is almost identical to that of the *apo-*structure with loss of coordination occurring to the Asp-267 residue, which has instead been mutated to an alanine. The M2 metal ion is similarly coordinated to Glu-122, His-188, and His-209, and it is striking that an additional interaction is formed with the carbonyl group of the GlcNAc6P substrate, replacing the water molecule in the *apo*-structure. The second chain of the co-crystal structure, where there is no GlcNAc6P substrate, can also be considered as another view of the *apo*-structure. Here there is a translational movement of His-134, which is located within the Glu-122–Pro-136 loop that blocks the active site, such that the histidine side-chain is 2.6 Å from the cadmium ion (Fig. 7). In the ligand-bound structure, this histidine residue is within 3 Å of the carbonyl oxygen of the substrate. This histidine is important in catalysis because its mutation in MMNagA (His-139) results in loss of activity (Table 1).

## Discussion

In prokaryotic species, it has been demonstrated that NagA is involved in the deacetylation of GlcNAc6P with an important role in carbohydrate metabolism and the recycling of murein fragments from the cell wall (11, 12, 34). A putative NagA enzyme, organized in an operon that differs from other bacterial organisms, has been identified in *Mtb* through genomic and proteomic studies and is predicted to be essential in the *Mtb* pathogen (17–19). Our biochemical and structural analyses of mycobacterial NagA enzymes are an important step to understanding amino-sugar assimilation, recycling, and metabolism in *Mtb*.

Our X-ray crystallographic structure determination reveals that MSNagA conforms to the urease structural superfamily, with the same overall fold, domain topology, and architecture as other NagA enzymes whose structures have been determined (21, 22, 24). MSNagA comprises two domains with formation of the active-site binding pocket occurring within domain I of the enzyme. Recruitment of a nonconserved Arg-219 from domain I from the adjacent monomer explains the dimeric nature of the enzyme. It is noteworthy that the mycobacterial NagA enzymes also form functional dimers. Mapping of Arg-219 to TBNagA reveals that this residue is instead replaced by a histidine residue, and it is probable that the invariant TBNagA arginine residue (Arg-220 MSNagA, corresponding to Arg-225 MMNagA and Arg-221 TBNagA) is utilized, along with the adjacent histidine residue that facilitates additional interactions with the 6-phosphate group of GlcNAc6P in a similar pincer manner to that of *B. subtilis* NagA (24). Modification of Arg-225 in MMNagA resulted in loss of catalytic function, indicating the important functional role of this conserved residue. Domain II appears to have no role in substrate binding, and the biological role of this small β-barrel/sandwich domain remains unclear. Our attempts to investigate the role of the β-barrel domain of MSNagA through the generation of a truncation mutant was hindered by the loss of production of soluble recombinant protein and suggests that the β-barrel domain has a role in the stability of MSNagA.

Importantly, in these studies we are able to unambiguously determine the position and orientation of the carbonyl group of GlcNAc6P, which is displaced in the NagA catalytic reaction. The binding mode of the biological GlcNAc6P substrate with MSNagA is very similar to that identified in the structure of *B. subtilis* NagA in complex with the GlcN6P product (24) and an *E. coli* NagA catalytically inactive mutant in complex with a *N*-methlhydroxyphosphinyl-d-glucosamine-6-phosphate transition state inhibitor (22). The MSNagA complex structure indicates that the active site does not undergo any major structural change on binding the GlcNAc6P substrate; however, two loop regions demonstrate a capacity for conformational flexibility and an importance in facilitation of substrate binding. A notable difference between mycobacterial NagA enzymes and NagA enzymes from other bacterial species is the presence of a cysteine at position 131 (MSNagA), which is replaced by a corresponding lysine residue in nonmycobacterial species and is located in the flexible loop that precludes the physiological substrate from binding. On the basis of the significant variation of a noncatalytic cysteine located within a region linked with substrate binding, there is the potential to exploit this reactive sulfhydryl moiety with the view to designing inhibitors specific for the TBNagA enzyme. A recent example of a cysteine-targeted therapeutic approach is in the development of irreversible peptidomimetic inhibitors to a noncatalytic cysteine located in the hepatitis C virus protease (35).

Our kinetic analysis reveals that MMNagA and MSNagA enzymes catalyze the deacetylation of the amino-sugar GlcNAc6P to form GlcN6P and acetate. Biochemical characterization has provided the first evidence of the function of mycobacterial NagA enzymes and demonstrated that MMNagA and MSNagA have comparable kinetic profiles to each other, and the kinetic constants reported here for MSNagA and MMNagA are comparable with the *E. coli* NagA enzyme in our fluorescent assay. The presence of a binuclear metal-binding site and two divalent metal ions located in each active site of MSNagA, which are both required for efficient catalysis and structural stability, is comparable with the Gram-positive NagA homolog and substantiates evidence that NagA enzymes have diverged with an evolutionary adaption that enables the mechanism of activation and stabilization of the carbonyl intermediate to differ depending on the metal requirements of the specific organism.

From the extensive panel of carbohydrates tested in this study, there was a clear preference of MMNagA and MSNagA for GlcNAc6P over the epimeric amino-sugar analogs ManNAc6P and GalNAc6P, indicating that the mycobacterial NagA enzymes are sensitive to the stereochemical requirements of the substituent at the C2 and C4 of the substrate. Although there is a clear preference for an equatorial *N*-acetyl group at C2, an axial C2 *N*-acetyl group, in the case of ManNAc6P, can be moderately tolerated. In contrast, there is a highly stringent selection for the equatorial C4 hydroxyl group of GlcNAc6P, with a marked reduction in the catalytic efficiency *k*_cat_ and an increase in the observed *K_m_* for GalNAc6P, where the C4 hydroxy group is positioned in an axial conformation. This was also observed for the *E. coli* NagA enzyme with the epimeric GalNAc6P substrate (23). Furthermore, the presence of a phosphate group at C6 is required for efficient catalysis to proceed. The biochemical results are consistent with the crystal structure of the complex and reveal that mycobacterial NagA enzymes are fine-tuned with a precise binding network that allows for the specific recognition and catalysis of the GlcNAc6P amino-sugar. Mammalian species have the ability to accommodate the larger *N-*glycolyl group of *N*-glycolylglucosamine-6-phosphate and deacetylate the acetyl and glycolyl GlcN6P analogs with equivalent efficiency (30). However, our studies demonstrate that mycobacterial NagA enzymes are distinct from these mammalian deacetylase enzymes and do not have this capacity to tolerate the introduction of a larger glycolyl group at the C2 position, indicating that mycobacterial NagA enzymes have evolved to have unique specificity and function.

The stringent recognition of the murein fragment GlcNAc6P over *N*-acetyl muramic acid 6-phosphate by mycobacterial NagA enzymes is interesting and may occur to ensure the integrity of *Mtb* peptidoglycan recycling and amino-sugar metabolism. *Mtb nagA* is encoded along with genes involved in peptidoglycan biosynthesis and carbohydrate uptake (Fig. 2) (17, 26), indicating the *Mtb* NagA is primed to play a role in the recycling of GlcNAc6P derived from the breakdown of cell-wall peptidoglycan. NagA has been shown to be involved in amino-sugar acquisition and peptidoglycan recycling in a number of other organisms including *E. coli* (36) (11), *B. subtilis* (37), *Staphylococcus aureus* (38), and *Streptomyces coelicolor* (34, 39). Hydrolysis of *Mtb* peptidoglycan is mediated through lytic transglycosylase resuscitation factors (40), and it is likely that *Mtb* has evolved functional pathways to enable the recovery of murein fragments within the nutrient poor macrophage environment. The *Mtb* solute-binding protein UspC of the UspABC transporter has recently been shown to be involved in the recognition of amino sugars with a potential role in recycling deacetylated PG fragments (41). Intriguingly, *Mtb* lacks the genetic machinery for phosphotransferase systems, and given the genomic organization of *nagA*, it is tantalizing to link the functional role of the *Mtb* SugI transporter to the import of phosphorylated carbohydrates, which subsequently acts in concert with the deactylase NagA enzyme to recycle and optimize the use of restricted carbohydrates during intracellular infection.

In conclusion, the NagA enzymes from *M. smegmatis* and *M. marinum*, with high sequence identity to the *M. tuberculosis* analog, are the first NagA enzymes to have been characterized both structurally and kinetically from mycobacteria. Given that there is almost identical sequence identity of these enzymes with the active-site of TBNagA, these mycobacterial homologs represent good model systems to understand the NagA enzyme from *Mtb*. Our data clearly indicate that mycobacterial NagA enzymes have a clear substrate preference for GlcNAc6P and other amino-sugar derivatives are poorly tolerated. Eukaryotic NagA enzymes convert GlcNAc6P directly to GlcNAc-1-phosphate, have low sequence identity (34%) to TBNagA, and differ in substrate selectivity. In this context, the conversion of GlcNAc6P to GlcN6P by mycobacterial NagA is a bacterial specific process that occurs at an essential metabolic chokepoint in the GlcN6P degradation pathway. Given the importance of TBNagA enzymes in the synthesis of essential mycobacterial cell wall components, TBNagA may serve as a novel molecular drug target, and these studies provide a framework to exploit this enzyme in the search for new TB therapeutic agents.

## Experimental procedures

### Materials and reagents

All chemicals and reagents were purchased from Sigma–Aldrich, unless specified, with the exception of all of the carbohydrates used in this study, which were purchased from Carbosynth. PCR and restriction enzymes were obtained from New England Biolabs. Double-distilled water was used throughout.

### Plasmid constructs, protein expression, and purification

Full-length GlcNAc-6-phosphate deacetylase (*nagA*) genes from *M. tuberculosis*, *M. marinum*, and *M. smegmatis* were amplified by PCR from the corresponding gDNA. The primer sequences are listed in Table S1. The PCR products were ligated into either NdeI and HindIII sites of the pET28a (+) (Novagen) for expression in *E. coli* or pYUB1062 (27) for expression in *M. smegmatis* mc^2^4517, resulting in the constructs *TBnagA_pet28a*, *MMnagA_pet28a*, *MSnagA_pet28a*, and *MSnagA_pYUB1062*. Targeted single-site or double-site substitutions were introduced into *MMnagA_pet28a* and *MSnagA_pet28a* using the primers (Table S1), with Phusion Polymerase and the PCR cycle (98 °C, 30 s; 25 cycles of 98 °C, 10 s; 60 °C, 30 s; 72 °C, 4 min; followed by 5 min at 72 °C), followed by digestion with 1 μl of DpnI. Plasmid sequences were verified by sequencing (GATC) and used for protein expression.

### Protein expression of NagA in E. coli

*E. coli* BL21(DE3) competent cells were co-transformed with the appropriate *nagA_pet28a* expression plasmid and the GroES 60.2 *Mtb* chaperone and grown at 27 °C to an optical density at 600 nm (*A*_600_) of 0.5 in terrific broth medium (Difco) supplemented with 50 μg ml^−1^ kanamycin and 100 μg ml^−1^ ampicillin. Protein production was induced with 0.5 mm isopropyl-β-thiogalactopyranoside, and the cultures were grown at 16 °C overnight with shaking (180 rpm). The cells were harvested (4,000 × *g*, 30 min, 4 °C) and resuspended in lysis buffer (20 mm Tris, 300 mm NaCl, 10% glycerol, pH 8.0 (buffer A)) supplemented with 0.1% Triton X-100 and frozen at −80 °C until further use.

### Protein expression of NagA in M. smegmatis

*M. smegmatis* mc^2^4517 electrocompetent cells were transformed with the appropriate *nagA_pYUB1062* construct and grown at 37 °C to an *A*_600_ value of 0.8 in LB medium supplemented with 0.05% Tween 80, 0.2% glycerol, 25 μg ml^−1^ kanamycin, and 100 μg ml^−1^ hygromycin. Protein production was induced with 0.2% acetamide, and the cultures were grown at 37 °C for an additional 20 h with shaking (180 rpm). The cells were harvested and resuspended in lysis buffer (buffer A) supplemented with 0.1% Triton X-100 and frozen at −80 °C until further use.

### Protein purification

Complete protease inhibitor mixture (Pierce), 5 mm MgCl_2_, 2 mg of DNase, and 20 mg of lysozyme were added to the resuspended pellet, and the pellet was either sonicated (*E. coli* pellets) on ice (Sonicator Ultrasonic Liquid Processor XL; Misonix) or passed through a cell disruptor at 4 °C (*M. smegmatis* pellets) (Constant Systems, 25 kpsi). Following centrifugation (27,000 *g*, 40 min, 4 °C) the supernatant was filtered (0.45-μm pore size) and loaded onto a pre-equilibrated HisPur Co^2+^-affinity resin (Thermo Scientific, Pierce). The column was washed with buffer A (5 column volumes), and the recombinant NagA protein was eluted from the Co^2+^ resin with increasing concentrations of imidazole. Fractions containing the protein were dialyzed at 4 °C for 12 h against buffer A, and either a second HisPur Co^2+^-affinity resin purification step was undertaken or fractions containing NagA protein were dialyzed against 20 mm Tris-HCl, 100 mm NaCl, 10% glycerol pH 8.0 (buffer B) at 4 °C for 12 h and applied to a HiTrap Q-column (1 ml; GE Healthcare Life Sciences) pre-equilibrated with buffer B and eluted with NaCl (0.1–1 m). Fractions containing NagA were pooled and purified further using size exclusion chromatography. Gel filtration experiments were carried out on a Superdex 200 16/60 column (GE Healthcare) using 20 mm Tris, 300 mm NaCl, 10% glycerol, pH 8.0. Fractions containing NagA were pooled, 0.03% DDM and 1 mm DTT were added, and the protein was concentrated to 5–10 mg/ml (Vivaspin 2; GE Healthcare) and stored at −80 °C. The identity of the proteins was confirmed by tryptic digest and nanoLC–electrospray ionization–MS/MS (WPH Proteomics Facility, University of Warwick).

### NagA assay

Concentrated MSNagA and MMNagA enzymes were dialyzed into 20 mm Bis-Tris, 300 mm NaCl, 10% glycerol, pH 7.0. The *E. coli* NagA enzyme was commercially available and obtained from NZYtech. The activity of the NagA enzyme was measured at 37 °C in an end point assay by following the production of the fluorescent product formed with fluorescamine and primary amines at λ_ex_ of 340 nm and λ_em_ of 460 nm. Unless otherwise stated, the reaction was carried out in a 96-well microtiter plate in 20 mm Bis-Tris, 300 mm NaCl, 10% glycerol, pH 7.0, in a total reaction volume of 50 μl. The reaction was initiated by the addition of substrate and terminated by the addition of 50 μl of 0.4 m borate buffer pH 10, 40 μl of 5 mm fluorescamine, and 50 μl of dimethylformamide to label the free amines and the production of fluorescence was monitored at λ_ex_ of 340 nm and λ_em_ of 460 nm (Tecan Infinite M200). The production of free amine was quantified with a glucosamine standard. For characterization of the kinetics of NagA substrate initial utilization rates of NagA catalysis were obtained. Kinetic parameters were calculated and analyzed using nonlinear regression analysis (GraphPad Prism, v7). All measurements were performed in triplicate. The impact of pH on activity was determined at 37 °C in the pH range 4.0 to 9.0 with GlcNAc6P (5 mm concentration). The following buffers were used: phosphate-citrate buffer, 300 mm NaCl, 10% glycerol (pH 4.0–7.0), 20 mm Bis-Tris, 300 mm NaCl, 10% glycerol (pH 6.0–7.0), and 20 mm Bis-Tris propane, 300 mm NaCl, 10% glycerol (pH 6.0–9.0).

### Affinity studies with MST

NagA protein was labeled using Monolith His-tag labeling kit RED–Tris–nickel–nitrilotriacetic acid in 50 mm Tris-HCl, pH 7.4, 150 mm NaCl, 10 mm MgCl_2_, 0.05% Tween 20, and a constant concentration of NagA (50 nm) was used. Carbohydrates were prepared in water in the concentration range 0–0.5 M. The samples were loaded into the MonoLite NT.115 standard treated capillaries and incubated for 10 min before analysis using the Monolith NT.115 instrument (NanoTemper Technologies) at 21 °C using 40% laser power and 20% LED power. The binding affinities were calculated with GraphPad Prism software. All experiments were carried out in triplicate.

### Circular dichroism

Purified NagA proteins (0.3 mg/ml) were dialyzed in 20 mm Tris, 100 mm NaCl, 10% glycerol, pH 8.0 buffer, transferred into a 1-mm path-length quartz cuvette, and analyzed on Jasco J-810 DC spectrometer from 198 to 260 nm. The spectra were acquired in triplicate and averaged after subtraction of the buffer background.

### Inductively coupled plasma MS (ICP–MS)

NagA proteins were diluted into 72% ultrapure HNO_3_ and heated to 70 °C for 30 min and then diluted with MilliQ deionized water to give a final concentration of 2% HNO_3_. The metal contents of the NagA samples were determined by ICP–MS (Agilent ICP–MS 7500cx) and by comparison to solutions of known metal concentrations using external calibration standards, which were prepared through a serial dilution of a single ppm stock mixture of zinc, cadmium, cobalt, copper, manganese, nickel, and iron in 2% nitric acid. External standards were prepared at 1, 2.5, 5, 10, 25, 50, 100, 250, 500, and 1,000 ppb. The masses of the isotopes detected were ^55^Mn, ^57^Fe, ^59^Co, ^63^Cu, ^60^Ni, ^66^Zn, and ^111^Cd. ^166^Er was used as an internal standard. Operating conditions were: plasma gas flow rate, 15.0 liters/min; auxiliary gas flow rate, 0.15 liter/min; nebulizer flow rate, 0.808 rps; and RF power, 1,550 W.

### Crystallization and structure determination

Crystals of NagA were grown initially by vapor diffusion in 96-well plates (Swiss-Ci), using a Mosquito liquid handling system (TTP LabTech) by mixing 1:2 volumes (150 nl) of concentrated NagA (10 mg/ml) with reservoir solution. NagA crystals grew within a week at 22 °C in 0.12 m monosaccharide mix (Morpheus, Molecular Dimensions), 0.1 m imidazole/MES, pH 6.5, 20% (v/v) PEG 500 MME, 10% (w/v) PEG 20,000 with the addition of 10 mm CdCl_2_ additive. For co-crystallization experiments, the D267A MSNagA was incubated with 5 mm GlcNAc6P and incubated at 4 °C for 30 min before crystallization in the same reservoir conditions. Crystals were flash frozen in liquid nitrogen prior to data collection.

### Structure determination

The X-ray diffraction data for both *apo*- and ligand-bound MSNagA crystals were collected at I04 and I24 beamlines of Diamond Light Source, respectively. *Apo*-MSNagA diffraction data were indexed, integrated, and scaled with XDS (42) through the XIA2 pipeline and the CCP4 suite of programs (43). Initial phases were obtained by molecular replacement using PHASER (44) with the NagA structure from *E. coli* (PDB code 2P50, chain A) as a search model. AutoBuild (45) was initially used for the model building followed by iterative cycles of alternating manual rebuilding in COOT (46), and reciprocal space crystallographic refinement with PHENIX-REFINE (47). The electron density in one of the flexible loop region (residues 275–300) was very weak, and the model building in this region was done manually in O (48). The ligand-bound MSNagA crystal data were also indexed, integrated, and scaled with XDS (42) through the autoPROC (49) pipeline. Initial phases were obtained by molecular replacement using PHASER (44) with the structure of the *apo* MSNagA (Chain A) as a search model, and the structure was refined as per the *apo* MSNagA. A feature enhanced 2*F*_o_ − *F*_c_ map (fem map) was calculated to enhance the fine details of the ligand density and the GlcNAc6P ligand was fitted into the electron density map. The Grade Web server (Global Phasing Ltd., http://grade.globalphasing.org/cgi-bin/grade/server.cgi)^3^ was used for the ligand restraint generation and optimization. The conformation of the GlcNAc6P ligand was validated with Privateer (50). The occupancy of the refined ligand was set at 0.75 because the associated density suggested that the site was not fully occupied. At this resolution it is difficult to distinguish zinc and iron ions in the electron density. Based on the ICP–MS results, we decided to model the metal ions as zinc. This resulted in B-factors of the metal ions similar to those of the surrounding residues. For one metal site in the D267A ligand-bound MSNagA structure, the residual density suggested a more electron-rich ion, and therefore cadmium was modeled at this position. In both the *apo-* and ligand-bound MSNagA structures, the interacting distances between the amino acids and the zinc ion at the active site were restrained to 2.1 Å, whereas no such restraints were applied to the cadmium ion interactions. Noncrystallographic symmetry restraints were initially used during refinement cycles; however, these were not used in the final refinements. Because of the higher resolution of ligand-bound MSNagA, it was used as a reference model for generating dihedral angles restraints during the refinement of *apo*-MSNagA.

The model of the *apo-*MSNagA structure determined comprises residues 1–377 in all chains (A–D), with an additional two residues defined (1–379) in chain B. MSNagA is a 385-amino acid protein, and clear density for the final C-terminal residues was not observed. The first residue of the partially ordered N-terminal His_6_ tag can also be observed in chains A–D. In the *apo*-MSNagA structure, there is one disordered region in chain D between residues 276 and 302. In chains A–C, weak electron density for this region was observed, indicating that this loop region of MSNagA is flexible. In comparison, the ligand-bound GlcNAc6P–MSNagA complex structure residues 1–378 (chain A) and residues 1–382 (chain B) are well defined by their electron density maps, enabling the positions of these amino acid residues to be determined with an additional three residues of the N-terminal His_6_-tag linker observed in both chains. Structure validations were done by MolProbity (51).

In the Ramachandran plot of the ligand-bound structure, there is only one serious outlier per chain. This is for His-209, one of the metal ligands. This residue forms part of a β-turn and coordinates Zn1. Phe-211, which is on the margin of the allowed zone is also in this region. The density is unambiguous in this region, and the homologs in the PDB file have similar outliers. In the *apo*-structure, some of the residues in the flexible loops are on the margins of the allowed regions in the Ramachandran plot (see above). The figures were drawn using PyMOL (PyMOL Molecular Graphics System, version 2.0; Schrödinger), except those showing electron density, which were made using CCP4 mg (52).

### Synthesis

NMR spectroscopy (^1^H, ^13^C) was conducted on either a Bruker DPX-300 or Bruker DPX-400 spectrometer, and all chemical shifts (δ) are given in ppm relative to the solvent reference. The data are recorded as follows: chemical shift (multiplicity (s for singlet, d for doublet, t for triplet, m for multiplet, and br for broad), coupling constant(s) *J* are quoted in Hz). Mass spectroscopy was obtained using a Bruker HCT Ultra machine. TLCs were performed on Merck silica gel 60 F-254 TLC sheets and were visualized by staining with 10% H_2_SO_4_ in ethanol followed by heating. Flash chromatography was carried out using Sigma–Aldrich technical grade silica gel (pore size, 60 Å; particle size, 40–63 μm) as the stationary phase.

### N-Glycolylglucosamine-6-phosphate

Glucosamine-6-phosphate (200 mg, 0.772 mmol), glycolic acid (59 mg, 0.772 mmol), benzotriazol-1-yl-oxytripyrrolidinophosphonium hexafluorophosphate (483 mg, 0.926 mmol), and triethylamine (222 μl, 1.54 mmol) were dissolved in dimethylformamide (50 ml) and MilliQ water (5 ml), and the reaction mixture was stirred at room temperature for 16 h. The reaction mixture was concentrated *in vacuo*, re-dissolved in water (30 ml), and extracted with chloroform (3 × 30 ml). The aqueous phase was separated and concentrated *in vacuo*. The solid residue was subsequently suspended in THF (50 ml), filtered, and then washed with THF (5 × 5 ml), chloroform (5 × 5 ml), acetone (5 × 5 ml), and methanol (5 × 5 ml). The residue was then dissolved in water (20 ml), acidified to pH 1.0 (0.1 m HCl), and concentrated *in vacuo*. The solid residue was suspended in THF (50 ml), filtered, and washed with chloroform (5 × 5 ml), acetone (5 × 5 ml), and ethanol (5 × 5 ml). The residue was then dissolved in water and concentrated *in vacuo* to give a brown solid (70 mg, 33%): ^1^H NMR (400 MHz, D2O): δ_ppm_ 5.15 (^1^H, d, *J* = 2.5 Hz, C^1^H α-anomer), 4.89 (^1^H, d, *J* = 8.0 Hz, C^1^H β-anomer), 4.06 (2H, s, C = OCH_2_), 3.41–4.17 (6H, m, C^2–5^H and C^6^H_2_). ^13^C NMR (75 MHz, D_2_O): δ_ppm_175.5, 175.0 (C = O), 94.7 (C^1^H β-anomer), 90.0 (C^1^H α-anomer), 74.6, 73.4, 70.5, 70.4, 69.5, 69.4 (C^3–5^H), 64.3, 60.8 (C^6^H_2_), 55.2, 56.2 (C^2^H), 44.6 (C = OCH_2_). ^31^P NMR (121 MHz, D_2_O): δ_ppm_ 0.45. *m*/*z* (ES^−^) C_8_H_15_NO_10_P expected 316.0439, found 316.0438 [M-H]^−^.

### Chemoenzymatic synthesis of N-acetyl muramic acid 6-phosphate

*Clostidium acetobutylicum* MurK was overexpressed and purified as described previously (53). The enzymatic reaction contained *N*-acetyl muramic acid (50 mm), ATP (67 mm), MgCl_2_ (10 mm), and MurK (10 μg) in 50 mm Tris-HCl, pH 8.0, and was incubated at 37 °C for 7 h. The product was purified by silica column chromatography (5:4:2:1 *n-*butanol/methanol/ammonium hydroxide 35%/H_2_O) to give the product as a white solid (63 mg). ^1^H NMR (400 MHz, D_2_O) δ_ppm_: 5.47 (^1^H maj, br s, anomeric CH), 4.65 (^1^H min, d, *J* = 9.0 Hz, anomeric CH), 4.40 (^1^H maj, q, *J* = 7.0 Hz, CHCH_3_), 4.23 (^1^H min, m, CHCH_3_), 3.46–4.13 (5H maj + 5H min, m, sugar ring), 2.00 (3H, s, C = OCH_3_), 1.28–1.35 (3H, m, CHCH_3_). ^13^C NMR (100 MHz, D_2_O) α-anomer: δ_ppm_181.8 (C = O), 174.4 (C = O), 95.7, 90.0, 80.1, 78.6, 78.2, 76.7, 72.0, 71.0, 63.4, 62.5, 55.8, 54.2 (sugar ring + CHCH_3_), 22.0 (C = OCH_3_), 18.7 (CHCH_3_). ^31^P NMR (161 MHz, D_2_O) δ_ppm_ 1.66. *m/z* (ES^−^) C_11_H_19_NO_11_P expected 372.0701, found 372.0703 [M-H]^−^.

### Accession codes

Coordinates and structure factors for MSNagA have been deposited in the Protein Data Bank under accession codes 6FV3 for the ligand-free MSNagA structure and 6FV4 for the GlcNAc6P-MSNagA co-crystal structure.

## Author contributions

M. S. A., C. M. F., C. S. G., K. S. M., B. G., A. D. C., and E. F. formal analysis; M. S. A., C. M. F., C. S. G., C. C., K. S. M., B. G., A. D. C., and E. F. validation; M. S. A., C. M. F., C. S. G., C. C., A. D. C., and E. F. investigation; M. S. A., A. D. C., and E. F. visualization; M. S. A., C. M. F., C. S. G., C. C., K. S. M., B. G., A. D. C., and E. F. methodology; M. S. A., C. S. G., A. D. C., and E. F. writing-original draft; M. S. A., C. M. F., C. S. G., C. C., K. S. M., B. G., A. D. C., and E. F. writing-review and editing; C. M. F., C. S. G., A. D. C., and E. F. supervision; E. F. conceptualization; E. F. funding acquisition; E. F. project administration.

## Supplementary Material

Supporting Information

## Footnotes

3 Please note that the JBC is not responsible for the long-term archiving and maintenance of this site or any other third party hosted site.

## Footnotes

2 The abbreviations used are:

*Mtb*: *M. tuberculosis*
GlcNAc6P: *N*-acetylglucosamine-6-phosphate
GalNAc6P: *N*-acetylgalactosamine-6-phosphate
ManNAc6P: *N*-acetylmannosamine-6-phosphate
GlcNAc6S: *N*-acetylglucosamine-6-sulphate
GlcN6P: glucosamine-6-phosphate
NagA: *N*-acetylglucosamine-6-phosphate deacetylase
MMNagA: NagA from *M. marinum*
MSNagA: NagA from *M. smegmatis*
TBNagA: NagA from *M. tuberculosis*
AG: arabinogalactan
PG: peptidoglycan
PDB: Protein Data Bank
TB: tuberculosis
ICP–MS: inductively coupled plasma MS
MST: microscale thermophoresis.
