# Supplementary material for: Structural and functional determination of homologs of the Mycobacterium tuberculosis N-acetylglucosamine-6-phosphate deacetylase (NagA)
Source: J Biol Chem. 2018 May 4;293(25):9770–83. doi: 10.1074/jbc.RA118.002597 (PMC6016474; doi:10.1074/jbc.RA118.002597)
Supplement: Supporting Information [file supp_293_25_9770__index.html]

Structural and functional determination of homologs of the Mycobacterium tuberculosis N-acetylglucosamine-6-phosphate deacetylase (NagA) — NagA from mycobacteria specific for GlcNAc6P — Structural and functional determination of homologs of the Mycobacterium tuberculosis N-acetylglucosamine-6-phosphate deacetylase (NagA) — NagA from mycobacteria specific for GlcNAc6P — Supporting Information 

# Structural and functional determination of homologs of the *Mycobacterium tuberculosis N*-acetylglucosamine-6-phosphate deacetylase (NagA)

## Supporting Information

- Supporting information - Supporting information
