# Supplementary material for: Structural and functional determination of homologs of the Mycobacterium tuberculosis N-acetylglucosamine-6-phosphate deacetylase (NagA)
Source: J Biol Chem. 2018 May 4;293(25):9770–83. doi: 10.1074/jbc.RA118.002597 (PMC6016474; doi:10.1074/jbc.RA118.002597)

Electronic Supplementary Material

Structural and functional determination of homologs of the *Mycobacterium tuberculosis* *N*-acetylglucosamine-6-phosphate deacetylase (NagA)

Mohd Syed Ahanger[1], Christopher M Furze[1], Collette S Guy[1,2], Charlotte Cooper[1], Kathryn S Maskew[1], Ben Graham[2], Alexander D Cameron[1], Elizabeth Fullam[1]*

From the [1]School of Life Sciences, University of Warwick, Coventry, CV4 7AL, United Kingdom; [2]Department of Chemistry, University of Warwick, Coventry, CV4 7AL, United Kingdom

*To whom correspondence should be addressed: Elizabeth Fullam, School of Life Sciences, University of Warwick, Coventry, CV4 7AL, United Kingdom; e.fullam@warwick.ac.uk; Tel. +44 (0)2476 574239

Supplementary Tables

Table S1: Oligonucleotides used for recombinant expression plasmids. Restriction recognition sites are in italics. Codon encoding the amino acid mutation is indicated in bold type. His-tag sequences are underlined

**Name**	**Use**	**Sequence (5’-3’)**
Mtb_nagA_F	Clone *Mtb* nagA into pET28a	AAAAAA*CATATG*ACCGTGCTCGGCGCCGACGCC
Mtb_nagA_R	Clone *Mtb* nagA into pET28a	AAAAAA*AAGCTT*TCAGCCCACCCGCCAGTCATCG
Msmeg_nagA_F	Clone *M. smegmatis* nagA into pET28a	AAAAAA*CATATG*CTGCTGACCGCCGACACCGTGC
Msmeg_nagA_R	Clone *M. smegmatis* nagA into pET28a	AAAAAA*AAGCTT*TCACACCGTGTGCGCCGC
Mmar_nagA_F	Clone *M. marinum* nagA into pET28a	AAAAAA*CATATG*ACCCTCATCTGTGCCGG
Mmar_nagA_R	Clone *M. marinum* nagA into pET28a	AAAAAA*AAGCTT*CTAGTCCTCGACGGCCTGCC
Msmeg_nagA_F	Clone *M. smegmatis* nagA into pYUB1062	CACCAA*CATATG*CACCATCATCATCATCATGTGCTGC TGACCGCCGACACC
Msmeg_nagA_R	Clone *M. smegmatis* nagA into pYUB1062	TATAAA*AAGCTT*TCACACCGTGTGCGCCGCGCCGGG
MtbnagA_5	Clone *Mtb* nagA into pYUB1062	CACCAA*CATATG*CACCATCATCATCATCATGTGACCG TGCTCGGCGCCGAC
MtbnagA_3	Clone *Mtb* nagA into pYUB1062	AAAAAA*AAGCTT*TCAGCCCACCCGCCAGTCATCG
MMar_E127A_5	Mutate MMNagA residue Glu127Ala	CAGGCATTCATCTG**GCG**GGCCCTTGGCTGAG
MMar_E127A_3	Mutate MMNagA residue Glu127Ala	CTCAGCCAAGGGCC**CGC**CAGATGAATGCCTG
MMar_H139A_5	Mutate MMNagA residue His139Ala	GCACGGTGCGGCGCA**GCC**GACCACACCCAAGTG
MMar_H139A_3	Mutate MMNagA residue His139Ala	CACTTGGGTGTGGTC**GGC**TGCGCCGCACCGTGC
MMar_R225A_5	Mutate MMNagA residue Arg225Ala	CCTGGGCCAC**GCC**GAGCCCGGGCC
MMar_R225A_3	Mutate MMNagA residue Arg225Ala	GGCCCGGGCTC**GGC**GTGGCCCAGG
MMar_D272A_5	Mutate MMNagA residue Asp272Ala	GTCGCATTGGTCACC**GCC**GCGATAGCCGCGGC
MMar_D272A_3	Mutate MMNagA residue Asp272Ala	GCCGCGGCTATCGC**GGC**GGTGACCAATGCGAC
MMar_H249A_5	Mutate MMNagA residue His249Ala	CCGACGGGGTA**GCC**GTCCATCCCGC
MMar_H249A_3	Mutate MMNagA residue His249Ala	GCGGGATGGAC**GGC**TACCCCGTCGG
MMar_QXN_5	Mutate MMNagA residue His56Q and His58N	GTTCGTCGACATC**CAG**GTG**AAC**GGCGGAGCGGGCG
MMar_QXN_3	Mutate MMNagA residue His56Q and His58N	CGCCCGCTCCGCC**GTT**CAC**CTG**GATGTCGACGAAC
MMar_AXA_5	Mutate MMNagA residue His56Ala and His58Ala	GTTCGTCGACATC**GCC**GTG**GCC**GGCGGAGCGGGCG
MMar_AXA_3	Mutate MMNagA residue His56Ala and His58Ala	CGCCCGCTCCGC**GGCC**CA**CGG**CGATGTCGACGAAC
Msmeg_D267A_5	Mutate MSNagA residue Asp267Ala	TTCGCTGATCACCGCGGCGATGGCCGCC
Msmeg_D267A_3	Mutate MSNagA residue Asp267Ala	GGCGGCCATCGCCGCGGTGATCAGCGAA

Table S2: ICP-MS analysis

	Expression host	Mn	Fe	Co	Ni	Cu	Zn	Cd
MMNagA	*E. coli*	0	50.18	41.98	1.138	0	30.43	1.565
MMNagA	*E. coli*	0	52.47	43.99	1.066	0	30.54	1.541
MSNagA	*E. coli*	0.0005	26.08	18.02	0.0009	0	11.05	1.543
MSNagA	*E. coli*	0.0007	27.88	19.1	0.0009	0	14.87	1.534
MSNagA	*M. smegmatis*	0	6.05	31.19	2.201	0	77.14	1.539
MSNagA	*M. smegmatis*	0	6.202	32.05	2.263	0	78.81	1.544

Concentrations are in parts per billion (ppb)

Table S3: MST binding constants

Enzyme	Expression host	Substrate	MST K_d (mM)
MMNagA	*E. coli*	GlcNAc6P	1.2 ± 0.05
MMNagA	*E. coli*	GalNAc6P	26.3 ± 6.9
MMNagA	*E. coli*	ManNAc6P	3.8 ± 0.3
MMNagA	*E. coli*	GlcNAc6S	nd
MMNagA	*E. coli*	GlcNAc	nd
MMNagA QXN	*E. coli*	GlcNAc6P	0.3 ± 0.01
MMNagA AXA	*E. coli*	GlcNAc6P	0.2 ± 0.02
MMNagA E127A	*E. coli*	GlcNAc6P	3.0 ± 0.06
MMNagA H139A	*E. coli*	GlcNAc6P	4.2 ± 0.2
MMNagA R225A	*E. coli*	GlcNAc6P	0.4 ± 0.07
MMNagA H249A	*E. coli*	GlcNAc6P	0.5 ± 0.02
MMNagA D272A	*E. coli*	GlcNAc6P	4.7 ± 0.3
MSNagA	*E. coli*	GlcNAc6P	nd
MSNagA D267A	*E. coli*	GlcNAc6P	0.8 ± 0.3
MSNagA	*M. smegmatis*	GlcNAc6P	nd
MSNagA	*M. smegmatis*	GalNAc6P	17.6 ± 2.5
MSNagA	*M. smegmatis*	ManNAc6P	nd
MSNagA	*M. smegmatis*	GlcNAc6S	nd
MSNagA	*M. smegmatis*	GlcN6P	18.2 ± 0.5

‘nd’ – not determined. SD are from three independent experiments.

Supplementary Figures

Fig. S1. Sequence alignment of NagA from *M. tuberculosis* with NagA homologues

The sequence alignment was generated using Clustal Omega (www.ebi.ac.uk/Tools/msa/clustalo/) and ESPript version 3. Identical residues are indicated by a red background, and conserved residues are indicated by red characters. The secondary structure elements of *M, smegmatis* NagA are shown above the sequences and the secondary structure elements of *B. subtilus* (pdb 2vhl) are shown below the sequences).

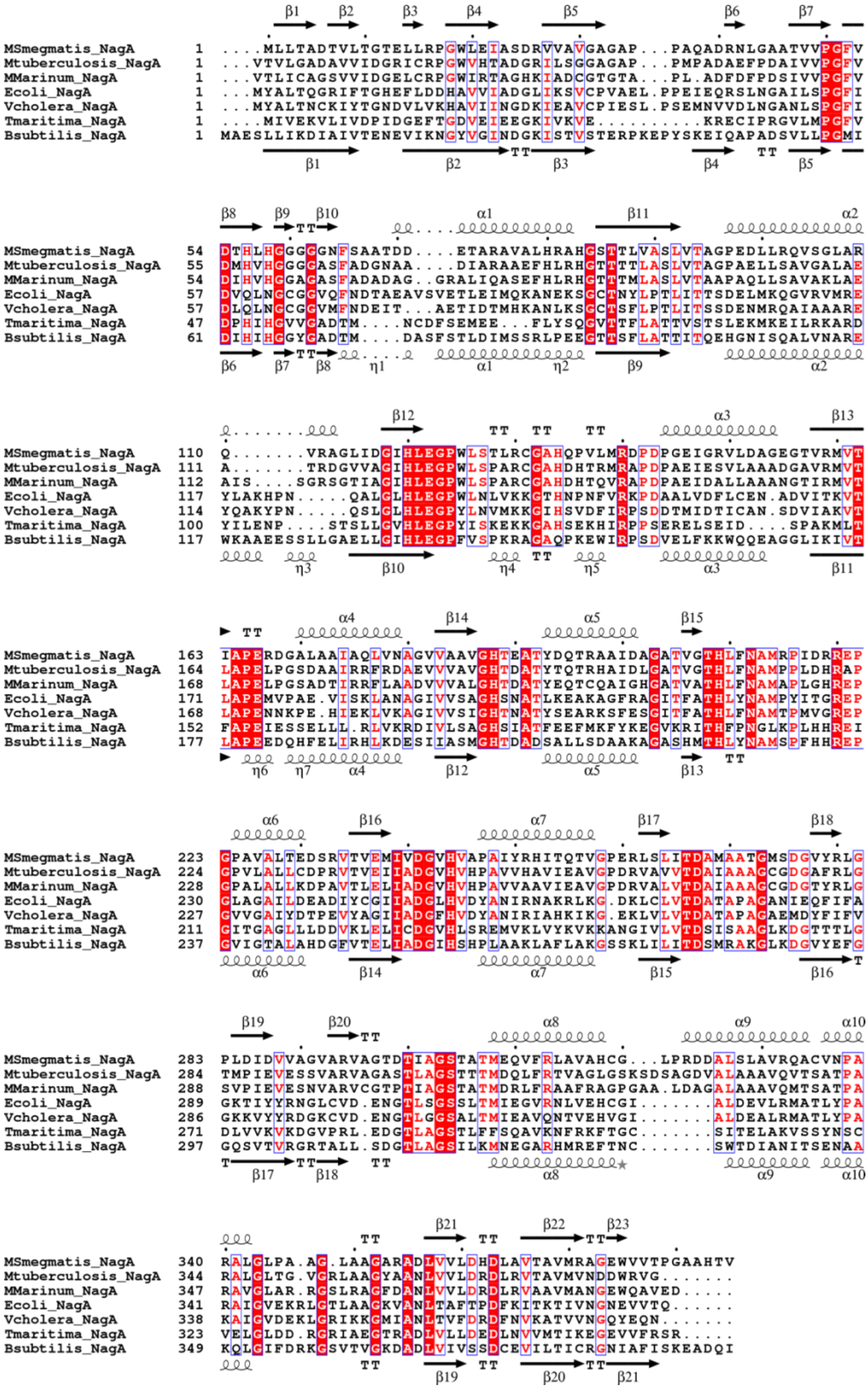

Fig. S2. SDS-PAGE analysis of the purification of *M. marinum* NagA from *E. coli*. A) Elution of His$_6$-tagged MMNagA from a Co^{2+} IMAC-column. Mr = molecular weight markers in kDa, WC = cell lysate, IS = insoluble fraction, S = soluble lysate, FT = flow through, W = wash, numbers of 0 - 1000 refer to the imidazole concentration in the elution buffer (units of mM). B) Second Co^{2+}-affinity purification: L= protein after dialysis. C) Size exclusion chromatography of MMNagA following anion exchange chromatography, with the volumes shown corresponding to figure S2D. D) Size exclusion trace with absorbance measured at 280nm. See Materials and methods for buffer compositions.

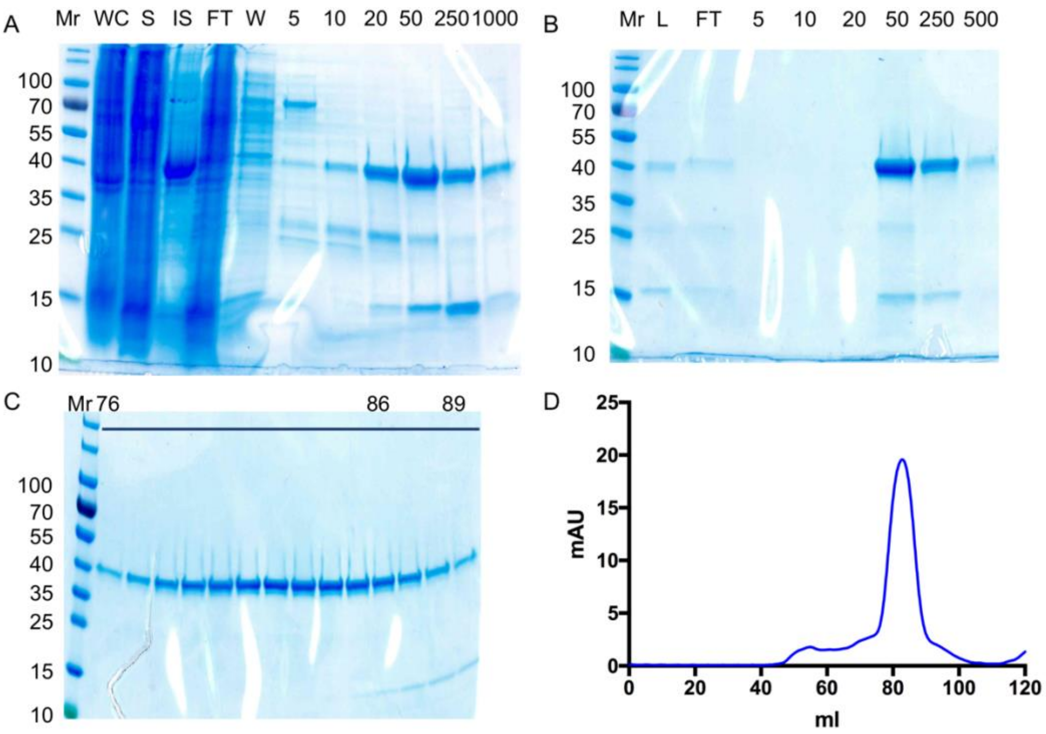

Fig. S3. SDS-PAGE analysis of the purification of MSNagA from *M. smegmatis*. A) Elution of His_6-tagged MSNagA from a Co^{2+} IMAC-column. Mr = molecular weight markers in kDa, WC = cell lysate, IS = insoluble fraction, FT = flow through, numbers of 0 to 1000 refer to the imidazole concentration in the elution buffer (units of mM). B) QHP anion exchange chromatography of MSNagA following the Co^{2+}-column purification step. FT = flow through, numbers across the top indicate NaCl concentration in the elution buffer (units of mM). C) Size exclusion chromatography of MSNagA following anion exchange chromatography, fractions collected from peak 1 of the gel filtration trace are shown. D) Size exclusion trace with absorbance at 280nm. See Materials and methods for buffer compositions.

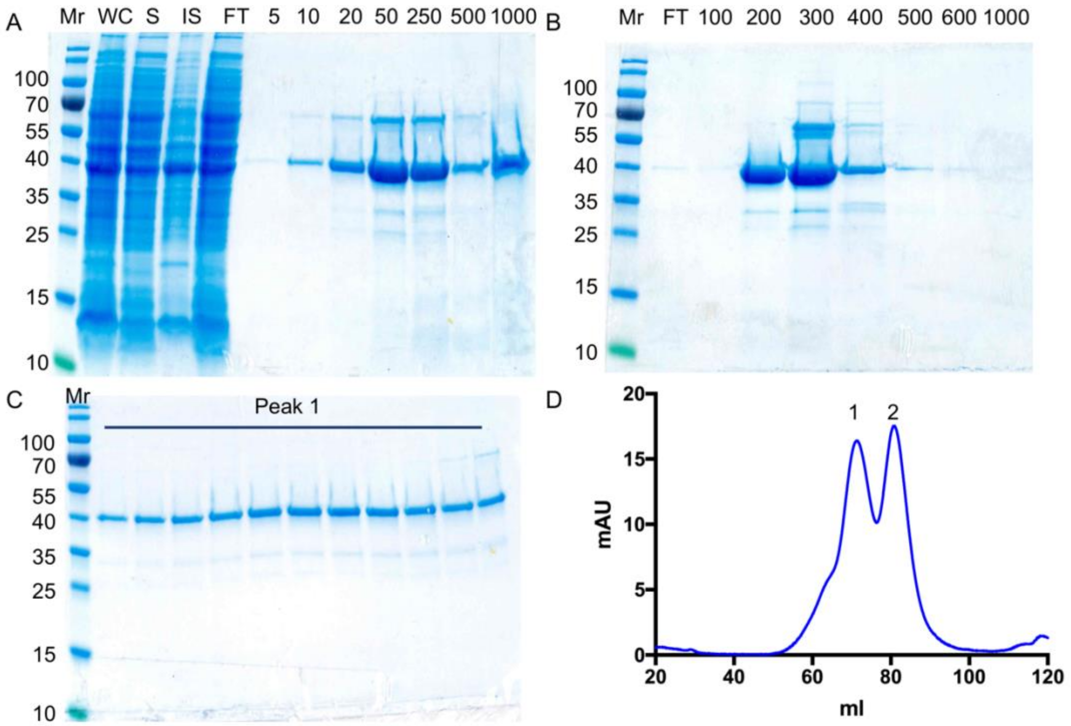

Fig. S4. CD spectra of MMNagA and MSNagA and site-directed mutant proteins

A) CD spectra of the MMNagA (green), MSNagA (blue), MMNagA E127A (red), MMNagA H139A (pink), MMNagA R225A (orange), MMNagA H249A (black), MMNagA D272A (brown), MMNagA QXN (dark green), MMNagA AXA (purple), MSNagA D267A (grey),

B) CD spectra of MMNagA (solid green line), MMNagA after treatment to remove the metal ion (green dotted line), MSNagA (solid blue line), MSNagA after treatment to remove the metal ion (blue dotted line), MSNagA after 3 days at 4 ^{o}C (blue dashes).

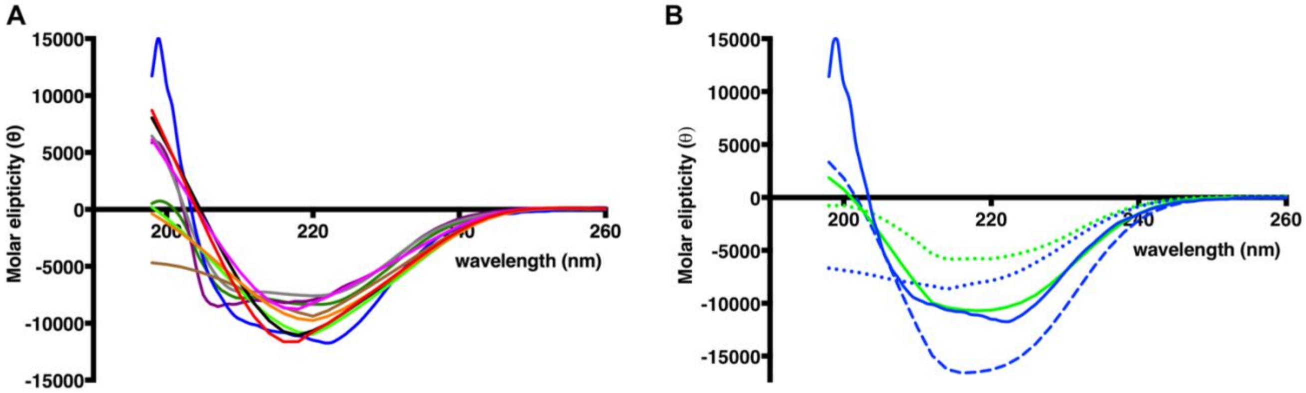

Fig. S5. pH dependence on MMNagA and MSNagA specific activity

A) The effect of pH on MMNagA activity; **B)** The effect of pH on MSNagA activity

Phosphate citrate buffer (black), bis-tris buffer (red), bis-tris propane buffer (blue). . Assay conditions are detailed in the materials and methods section. SDs are determined from three independent experiments.

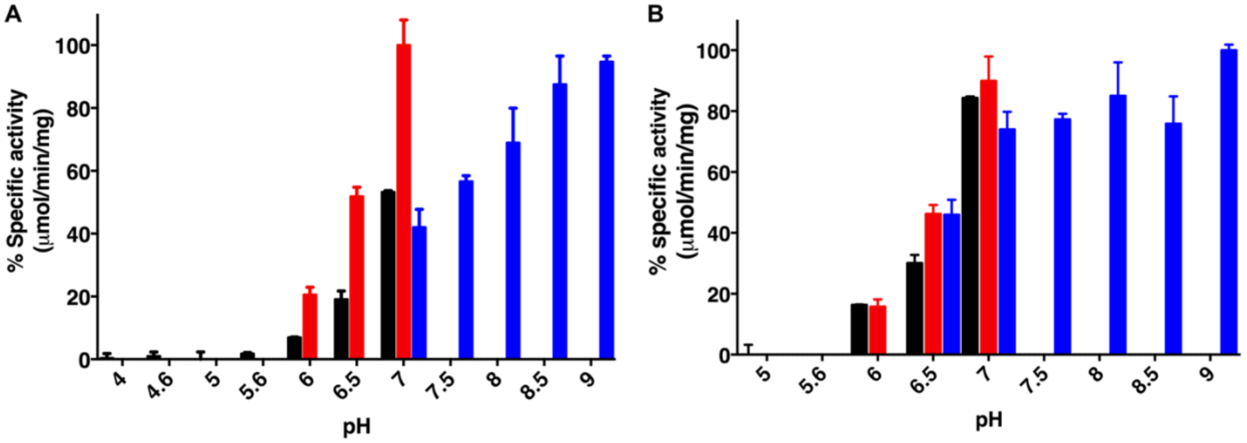

Fig. S6 Electron density for the GlcNAc6P substrate

Electron density map contoured at 0.44 electrons/Å^3. Carbon atoms are shown in green, oxygen atoms are shown in red, nitrogen atoms in blue and phosphate atom in purple. The figure was prepared using CCP4mg. The .mtz file was loaded directly with the default settings and clipped to select for the GlcNAc6P atoms.

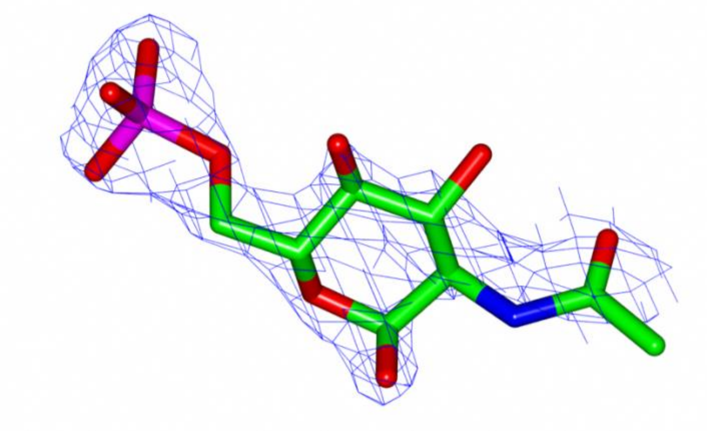

Fig. S7 Changes associated with lid opening and ligand binding. Superposition of the ligand bound protein (chain A, blue) with the non-ligand bound protein (chain B, grey). The GlcNAc6P ligand is shown in green. Cys131 is selected and shown in stick format (cyan in ligand bound – chain A, grey in non-ligand bound - chain B).

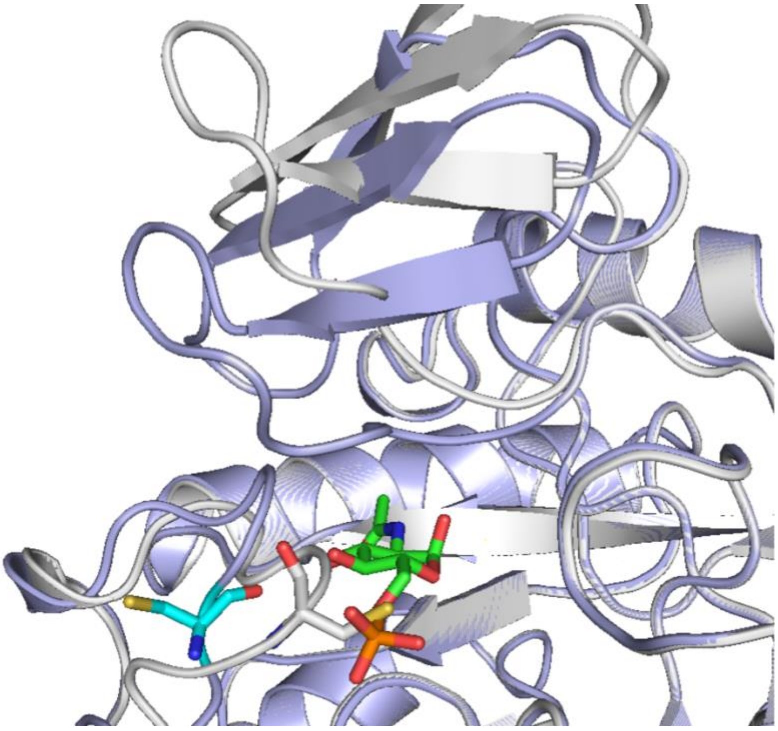

Fig. S8 Comparison of the interaction with the conserved arginine residue from the adjacent molecule. Superposition of the ligand-bound MSNagA structure with the NagA from *B. subtilus* (pdb 2vhl). MSNagA (chain A, light blue, text label black; chain B, dark blue, text label red) and *B. subtilus* NagA (cyan). GlcNAc6P from MSNagA is shown with green carbon atoms, the MSNagA metal ions are shown in grey spheres. Selected residues are shown in stick format, coloured by chain.

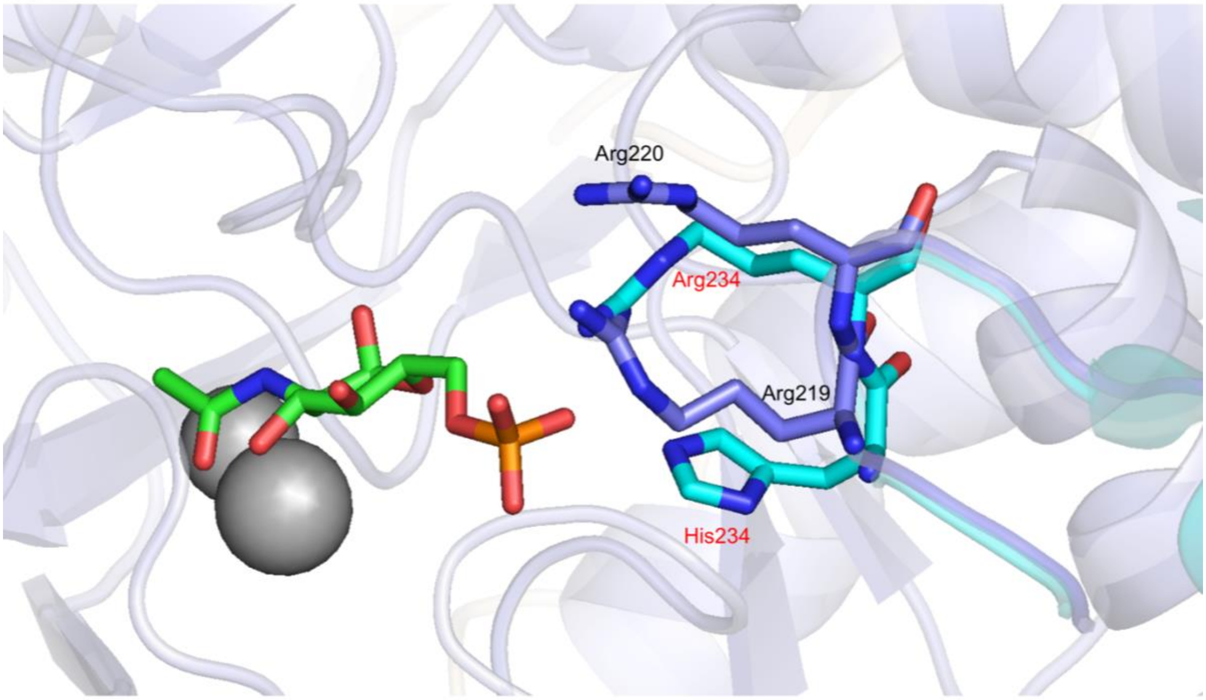

Fig. S9 Electron density map for the metal binding sites. A) Illustration showing 2mFo-DFc map (contoured at 1.0 σ) of the metal binding site in the *apo*-MSNagA structure (Chain A, magenta). Zn^{2+} silver spheres, H_2O red spheres and selected amino acid residues in stick representation (magenta). B) Illustration showing 2mFo-DFc map (contoured at 1.0 σ) of the metal binding site in the ligand-bound MSNagA structure (chain A, yellow), Zn^{2+} silver spheres, Cd^{2+} orange sphere, Cl^- green sphere, GlcNAc6P carbon atoms coloured green and selected amino acid residues in stick representation (yellow). Figure prepared with PyMol.

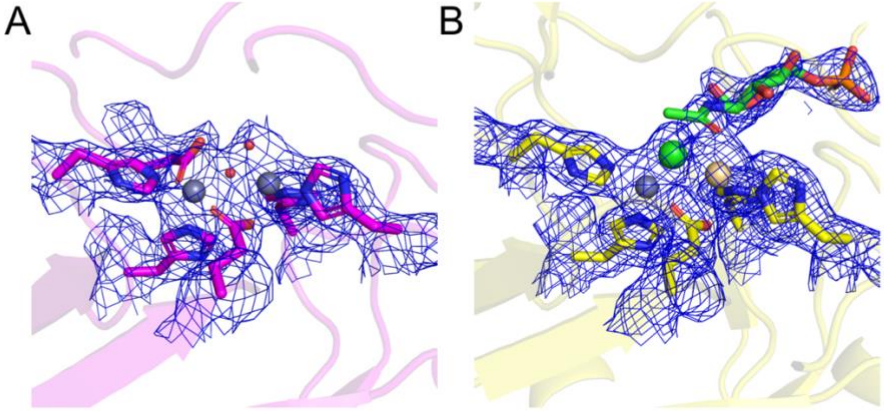

Supplement: Supporting Information [file supp_RA118.002597_136254_2_supp_132438_p87x4l.pdf]
